# Recent Advancements in the Characterization of D‐Amino Acid and Isoaspartate Post‐Translational Modifications

**DOI:** 10.1002/mas.21916

**Published:** 2024-11-18

**Authors:** Samuel Okyem, Jonathan V. Sweedler

**Affiliations:** ^1^ Department of Chemistry, Beckman Institute for Advanced Science and Technology University of Illinois at Urbana‐Champaign Urbana Illinois USA

**Keywords:** D‐amino acids, isoaspartate, mass spectrometry, peptides

## Abstract

One of the great triumphs of mass spectrometry‐based peptide and protein characterization is the characterization of their modifications as most modifications have a characteristic mass shift. What happens when the modification does not change the mass of the peptide? Here, the characterization of several peptide and proteins modifications that do not involve a mass shift are highlighted. Protein and peptide synthesis on ribosomes involves L‐amino acids; however, posttranslational modifications (PTMs) can convert these L‐amino acids into their D‐isomers. As another example, nonenzymatic PTM of aspartate leads to the formation of three different isomers, with isoaspartate being the most prevalent. Both modifications do not alter the mass of the peptide and yet can have profound impact on the physicochemical characteristics of the peptide. Several MS and ion mobility techniques are highlighted, as are other methods such as chromatography, enzymatic enrichment, and labeling. The challenges inherent to these analytical methods and prospective developments in bioinformatics and computational strategies are discussed for these zero‐dalton PTMs.

## 1 Introduction

After performing a high‐quality LC‐MS based bottom‐up proteomics experiment, an observant individual may notice a few cases where the same peptide is assigned to two distinct retention time peaks. If the retention times differ significantly, one can ask how this is possible? What properties caused the two peptides to separate in an LC run but to have the same assigned sequence? While there are several instrumentation possibilities (poor injections, bad connections, overloaded column), let's assume the analytical performance is fine. Then what?

Obvious possibilities include similar (scrambled) sequences and peptides with different locations of PTMs. For example, consider a peptide with a single phosphorylation that has three possible locations—each of the three possibilities is a distinct molecule and may separate from each other. A more insidious possibility includes a labile modification such as sulfation; a peptide with sulfation may separate from the unmodified version during chromatography and the sulfation may then be eliminated during the ESI process (Myers et al. 1993; Yu et al. 2007). In this case, both peaks actually are the same within the mass analyzer. Positional isomers formed by amino acid rearrangement and differential acetylation can typically be resolved using tandem MS and Ion mobility spectrometry (IMS). However, the identification and characterization of sulfated peptides are particularly challenging due to the labile nature of the sulfate group (Önnerfjord, Heathfield, and Heinegård 2004). Traditional MS techniques often result in the loss of sulfation under typical ionization and fragmentation conditions, making it difficult to accurately identify sulfated peptides. Recent advancements have focused on mitigating this issue through innovative chemical and enzymatic modification strategies (Lietz et al. 2023). One such method involves the use of succinimidyl acetate to acetylate non‐sulfated tyrosine residues before MS analysis (Yu et al. 2007). This approach allows for the indirect identification of sulfated residues by detecting non‐acetylated tyrosine residues. The non‐acetylated tyrosine signals the presence of sulfation, as sulfated tyrosine does not react with succinimidyl acetate. In other words, there are solutions for these types of issues.

The presence of proline cis‐trans isomers within a peptide sequence can also affect chromatographic behavior, often leading to the appearance of split or multiple eluting peaks. This phenomenon is particularly pronounced when multiple proline residues are located close to the sequence, as the distinct conformational states of cis and trans isomers exhibit different physicochemical properties (Rusconi et al. 1985).

**Scheme 1:**
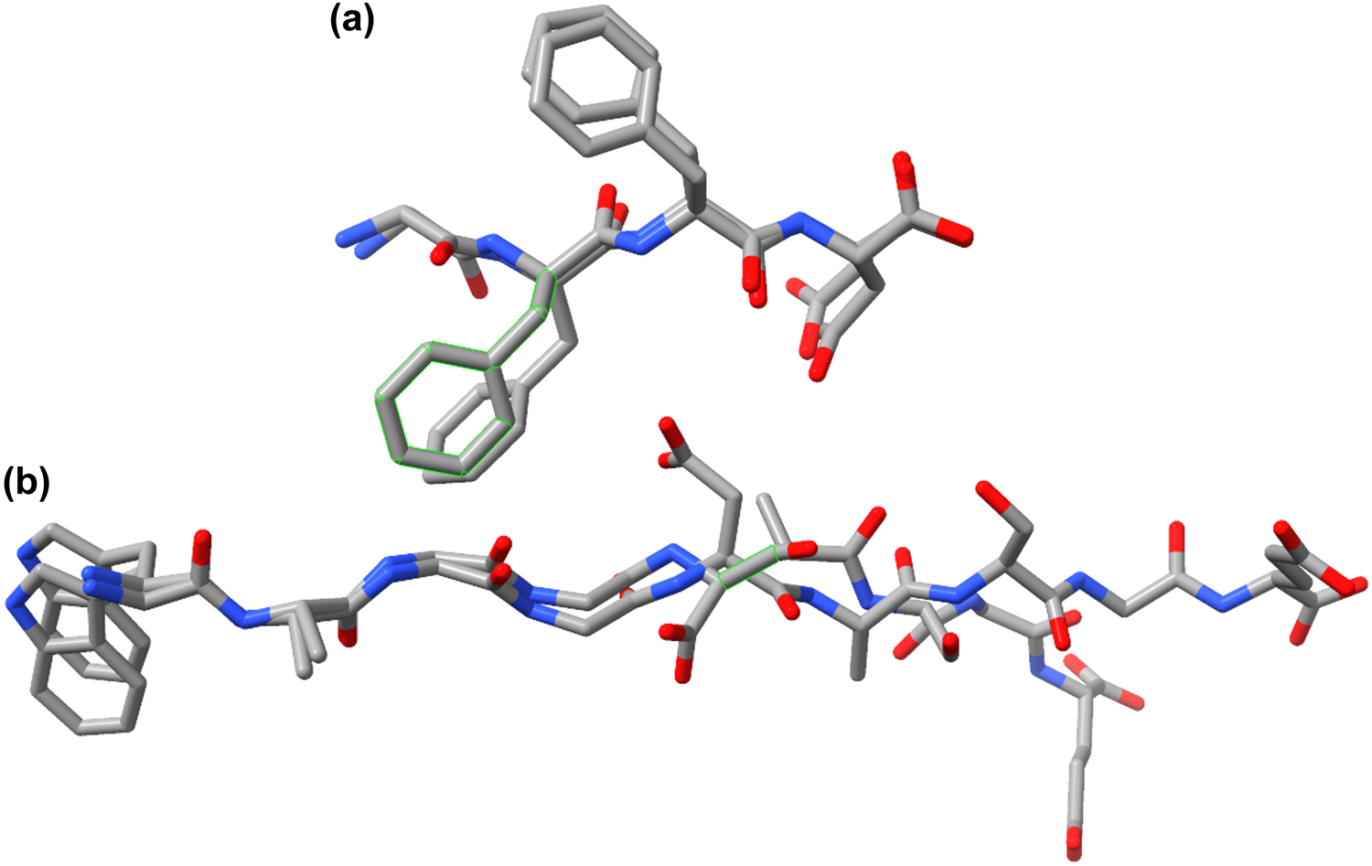
Overlay of a 3D computational model of (a) IACP (WAGGisoDASGE) & all‐L isomer, and (b) DAACP (GdFFD) & all‐L isomer. D‐phenyl alanine residue and methylene insertion are highlighted in green in a & b, respectively. The PDB files were created using PeptideConstructor python package and further modified/visualized in UCSF ChimeraX. [Color figure can be viewed at wileyonlinelibrary.com]

In this review, we highlight two other less well‐known modifications that do not result in a mass change but can have a pronounced effect on the 3D structure (Scheme 1). Bioactive peptides derived from preprohormone precursors undergo multiple posttranslational modifications (PTMs), including the conversion of specific L‐amino acids to D‐amino acids (Bai, Sheeley, and Sweedler 2009; Fricker 2008; Mast, Checco, and Sweedler 2020, 2021; Pan et al. 2005; Sossin, Fisher, and Scheller 1989) and the nonenzymatic isomerization of aspartate to isoaspartate (IsoAsp) (Shimizu et al. 2000, 2005; J. Wang et al. 2022). These modifications give rise to D‐amino acid‐containing peptides (DAACPs) and IsoAsp‐containing peptides (IACPs), respectively, which are classified as zero‐dalton PTMs due to their lack of mass change. D‐amino acids in peptides notably enhance stability against enzymatic degradation and can significantly increase biological activity (Bai et al. 2013; Checco et al. 2019). For instance, the neuropeptide GdFFD, featuring D‐phenylalanine, regulates feeding behaviors in *Aplysia californica* by engaging an achatin‐like receptor, whereas its all‐L counterpart is inactive. This peptide also shows remarkable stability in physiological pH and resistance to peptidase activity. (Bai et al. 2013) While DAACPs have been identified as signaling molecules and toxins in various metazoans (Mast, Checco, and Sweedler 2021), their endogenous presence in mammals remains unconfirmed. However, L‐to‐D isomerase activity has been detected in rat lungs, spinal cord, and cerebellum tissues (Andersen et al. 2024; Koh et al. 2010; Torres et al. 2007). This points to a potential but unexplored presence of DAACPs in mammals.

The accumulation of IsoAsp in proteins can lead to both beneficial and adverse effects, as seen in its role in protein degradation and fibrillization (Akbey and Andreasen 2022; Aswad, Paranandi, and Schurter 2000; Chatterjee et al. 2020; Johnson et al. 1989; Maji et al. 2009; Shimizu, Matsuoka, and Shirasawa 2005; J. Wang et al. 2022, 2023; Yamamoto et al. 1998). IsoAsp residues have been found in α‐crystalline of the aging human lens (Fujii et al. 1994; Hooi and Truscott 2011) and in proteins associated with cognitive declines, such as Aβ42/Aβ40, phosphorylated tau, and glial fibrillary acidic protein (Shimizu et al. 2002; Sugiki and Utsunomiya‐Tate 2013; Warmack et al. 2019). Elevated IsoAsp levels are also present in the serum albumin of Alzheimer's patients (J. Wang et al. 2021, 2023). IsoAsp is implicated in the functionality of bacterial biofilms and amyloid formation in peptide hormones (Akbey and Andreasen 2022; Maji et al. 2009).

Despite significant progress in peptide and protein‐based therapeutics over the past decades, challenges remain in maintaining stability against proteolytic and nonenzymatic degradation. IsoAsp accumulation in antibodies, for example, can lead to their degradation (L. Wang et al. 2022). The recent discovery of L/D isomerase activity in mammalian tissues (Andersen et al. 2024) introduces a new layer of complexity in understanding therapeutic peptide stability.

The physiological impact posed by the occurrence of DAACPs and IACPs underscores the need to develop robust analytical tools to investigate these modifications. Current strategies for analyzing these zero‐dalton PTMs primarily rely on tandem MS techniques that can produce diagnostic peaks or differentially abundant fragment ions for each isomer (DeGraan‐Weber, Zhang, and Reilly 2016; Mast, Checco, and Sweedler 2021; H. T. Wu, Van Orman, and Julian 2024). Liquid chromatographic techniques are also employed to separate peptide isomers based on their physicochemical properties (Livnat et al. 2016; Maeda et al. 2015; Winter, Pipkorn, and Lehmann 2009). Ion mobility spectrometry (IMS) can separate peptide isomers based on their size, shape, and charge, providing an additional dimension of separation that complements chromatographic and MS techniques (Jeanne Dit Fouque et al. 2017; Li, Delafield, and Li 2020; Q. Wu et al. 2020). Enzymatic enrichment and labeling can also facilitate the identification of peptide diastereomers (Kameoka 2003; Livnat et al. 2016; Silzel, Lambeth, and Julian 2022).

This review explores recent advances in analytical methodologies for detecting and differentiating DAACPs and IACPs. We focus on the integration of tandem MS and IMS techniques, which have enhanced the analysis of peptide diastereomers despite the inherent challenges posed by their identical mass. The review also discusses enzymatic strategies for the enrichment and labeling of these modified peptides, highlighting the critical need for continued development in this field to better understand and exploit the physiological roles of these unique PTMs.

## 2 MS Approaches for the Identification of DAACPs and IACPs

### 2.1 MS2‐Based Method for Identification of Isomerized Residues

Mass spectrometry is an essential analytical tool for characterizing a wide array of molecules, including peptides, proteins, lipids, carbohydrates, metabolites, and drugs (Bantscheff et al. 2012; Dettmer, Aronov, and Hammock 2007; King et al. 2023; Pukala and Robinson 2022). It provides detailed molecular information about analytes, although it requires a difference in mass to effectively characterize complex mixtures. This requirement complicates the analysis of isobaric species, such as peptide isomers. Various fragmentation methods have been developed to address this issue, particularly for peptide isomers arising from L‐D isomerization and isoAsp formation (C. M. Adams et al. 2004; C. M. Adams and Zubarev 2005; Bashyal et al. 2023; Chan, Chan, and O'Connor 2010; Edwards et al. 2022; O'Connor et al. 2006; H. T. Wu et al. 2023). The isomerization from aspartate to IsoAsp involves the insertion of a methylene group into the peptide backbone, enabling the generation of distinctive diagnostic fragment ions through different fragmentation techniques (Chan, Chan, and O'Connor 2010; DeGraan‐Weber, Zhang, and Reilly 2016; Lyon, Beran, and Julian 2017).

**Figure 1:**
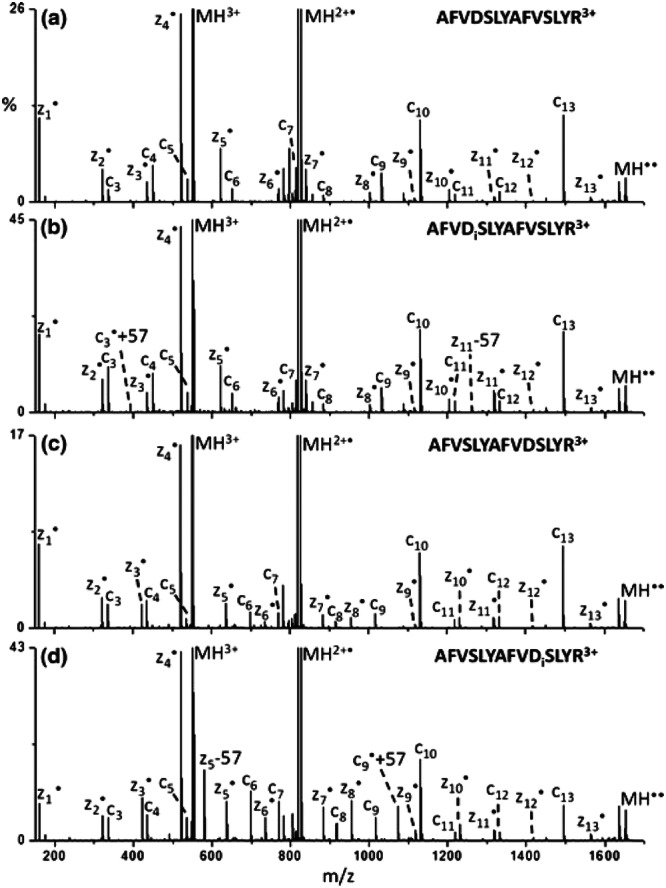
ETD mass spectra for triply charged (a) AFVDSLYAFVSLYR, (b) AFVDiSLYAFVSLYR, (c) AFVSLYAFVDSLYR and (d) AFVSLYAFVDiSLYR precursor ions. Diagnostic peaks at Z11‐57 and C*_9_ + 57 confirmed isoAsp in AFVDiSLYAFVSLYR and AFVSLYAFVDiSLYR, respectively. Figure adapted from DeGraan‐Weber et al.

These techniques include Collision‐Induced Dissociation (CID), Electron Capture or Transfer Dissociation (ECD/ETD), Free‐Radical Initiated Peptide Sequencing (FRIPS), and charge tagging. They can produce specific isoAsp diagnostic peaks such as y‐46, z‐57, c + 57, and b + H2O. The Reilly group has conducted studies to evaluate the effectiveness of these methods in localizing and identifying isoAsp residues. Their findings indicate that ETD is the most effective method for identifying isoAsp residues in high‐mass peptides (Figure 1). Meanwhile, charge tagging, specifically tagging peptides with o‐TEMPO‐Bz‐NHS followed by photodissociation, was successful in generating effective b + H_2_O diagnostic peaks for low‐mass peptides containing isoAsp (DeGraan‐Weber, Zhang, and Reilly 2016). Nonetheless, ETD requires multiply charged precursors to generate quality fragment ions for identification (Chan, Chan, and O'Connor 2010).

The Jackson and Julian group utilized Charge Transfer Dissociation (CTD) to identify and distinguish aspartate isomers in peptides (Edwards et al. 2022). In this technique, a kiloelectronvolt beam of helium cations abstracts an electron from an ionized peptide, forming a radical ion that enhances radical fragmentation. This method produces backbone and sidechain fragments, providing the molecular details necessary to differentiate isomers. Aspartate isomers are identified by comparing the relative abundance of fragment ions between each isomeric form.

The chirality recognition factor, R_chiral_, is defined as R_chiral_ = Rd/Rl, where Rd and Rl are the ratios of the most differing fragment ion pair between each peptide isomer. A significant variation in the relative abundance of fragment ions among peptide epimers can occur due to differences in dissociation's gas‐phase transition state energies (Jansson 2018; Tao, Quebbemann, and Julian 2012; H. T. Wu, Van Orman, and Julian 2023, 2024). These differences affect the abundance of fragment ions derived from each epimer and are instrumental for identifying isomerized residues within a peptide. When the R_chiral_ value exceeds 1, it suggests the presence of isomeric pairs, with higher values indicating a greater likelihood of successful chiral recognition. This chiral recognition factor is widely used by research groups to detect and confirm the presence of isomeric pairs in peptides (Bai, Sheeley, and Sweedler 2009, 2011; Lyon, Beran, and Julian 2017; H. T. Wu et al. 2023).

In Radical Directed Dissociation (RDD), the efficiency of fragment ion generation depends on the precursor ion's molecular structure. This specificity makes RDD more effective at identifying and distinguishing peptide isomers than other fragmentation methods. In this technique, peptides are first modified with a chromophore containing a carbon‐iodine (C‐I) bond. These modified peptides are then subjected to photodissociation (PD) by exposure to UV laser radiation at approximately 266 nm, which breaks the C‐I bond and forms the radical. This radical subsequently undergoes collisional activation to produce fragment ions (Lyon, Beran, and Julian 2017).

**Figure 2:**
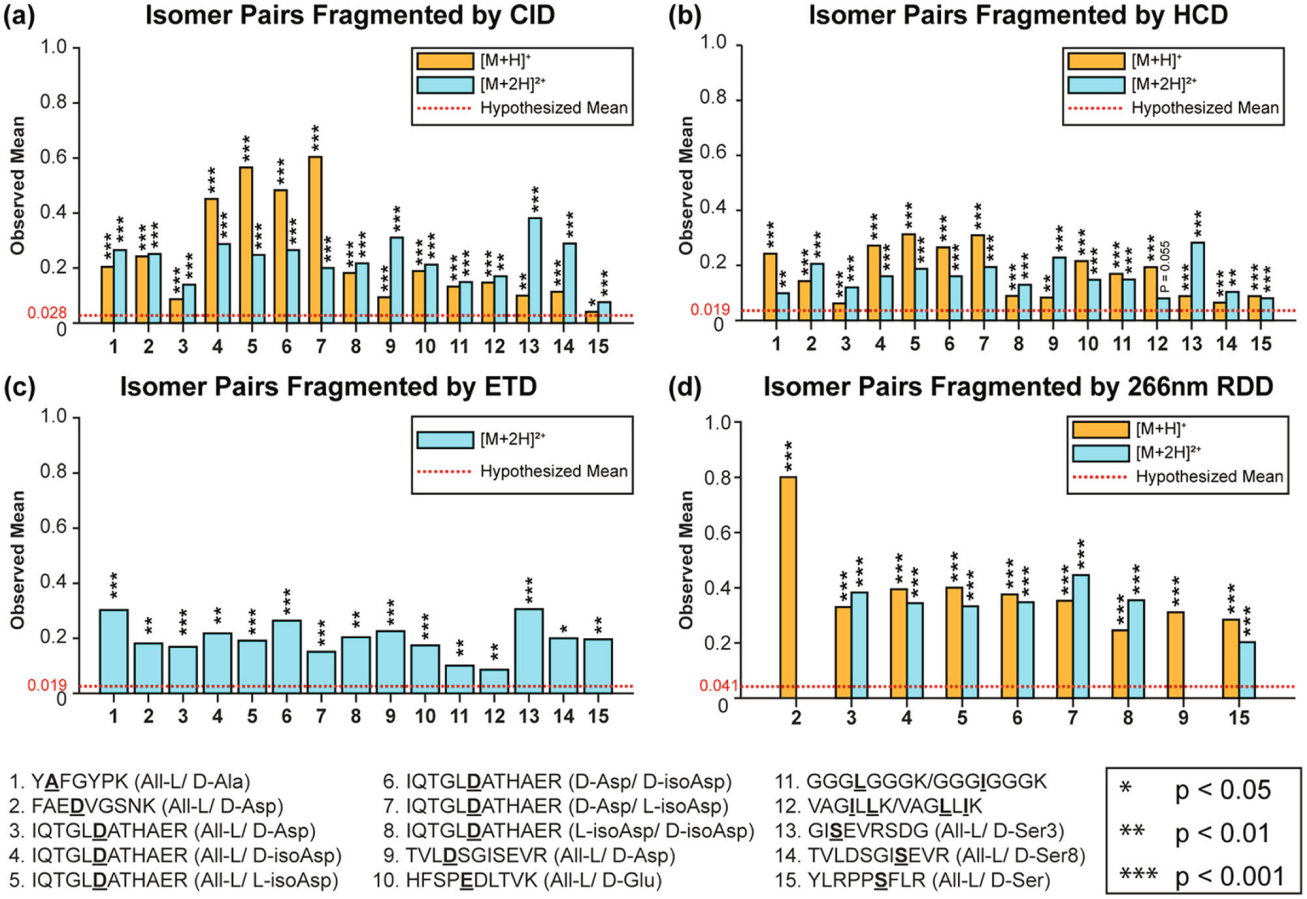
Differentiating peptide isomers by a variety of MS2 methods. The bar plot shows the calculated means for a variety of peptide isomers. P‐values were determined by a one‐sample *t*‐test against a hypothesized mean (red dotted line); hypothesized mean for each fragmentation method, and the data set was used as the average plus three standard deviations. Results are binned by charge state and fragmentation method (a) CID, (b) HCD, (c) ETD, and (d) RDD. All isomer pairs can be differentiated by any fragmentation method (*p < 0.05, **p < 0.01, ***p < 0.001). Isomerized residues are formated in bold and underlined. Figure Adapted from H. T. Wu et al. (2023) with permission from the American Chemical Society. [Color figure can be viewed at wileyonlinelibrary.com]

Although the R_chiral_ effectively discriminates between peptide isomers, it traditionally uses only two fragment ions from each epimer (Bai, Romanova, and Sweedler 2011; Mast, Checco, and Sweedler 2021; Tao and Julian 2014; H. T. Wu et al. 2023) leaving out other fragments that may contain valuable information for isomeric differentiation. To overcome this limitation, the Julian group developed a comprehensive statistical method that incorporates all fragment ions to differentiate and identify isomeric pairs (Figure 2) (H. T. Wu et al. 2023). This method involves acquiring multiple MS2 scans for each peptide isomer and calculating each fragment's fractional abundance by dividing the fragment's peak height by the total peak sum in the spectrum. Using statistical tests such as the *t*‐test, they could distinguish peptide isomers using conventional fragmentation techniques, including CID, HCD, ETD, and RDD. The differentiation is achieved by comparing the average fragment ions abundance from multiple MS2 scans for each isomer.

The dynamic exclusion window employed in data‐dependent acquisition precludes peptides of the same mass from being selected for fragmentation for a defined time, which may impact isomer identification. Although data‐independent acquisition (DIA) methods can be useful in addressing this challenge, the data analysis tools employed in DIA employ algorithms that select the best peak for a specific m/z, excluding other peaks that may be potential isomeric forms of the peptide. The Julian group has developed a data analysis pipeline that can enable the identification of isomers from a DIA data set (Hubbard et al. 2022). They successfully employed this approach to identify and quantify the levels of isoaspartate in tau protein.

### 2.2 Localization of Isomerized Residues by MS3

**Figure 3:**
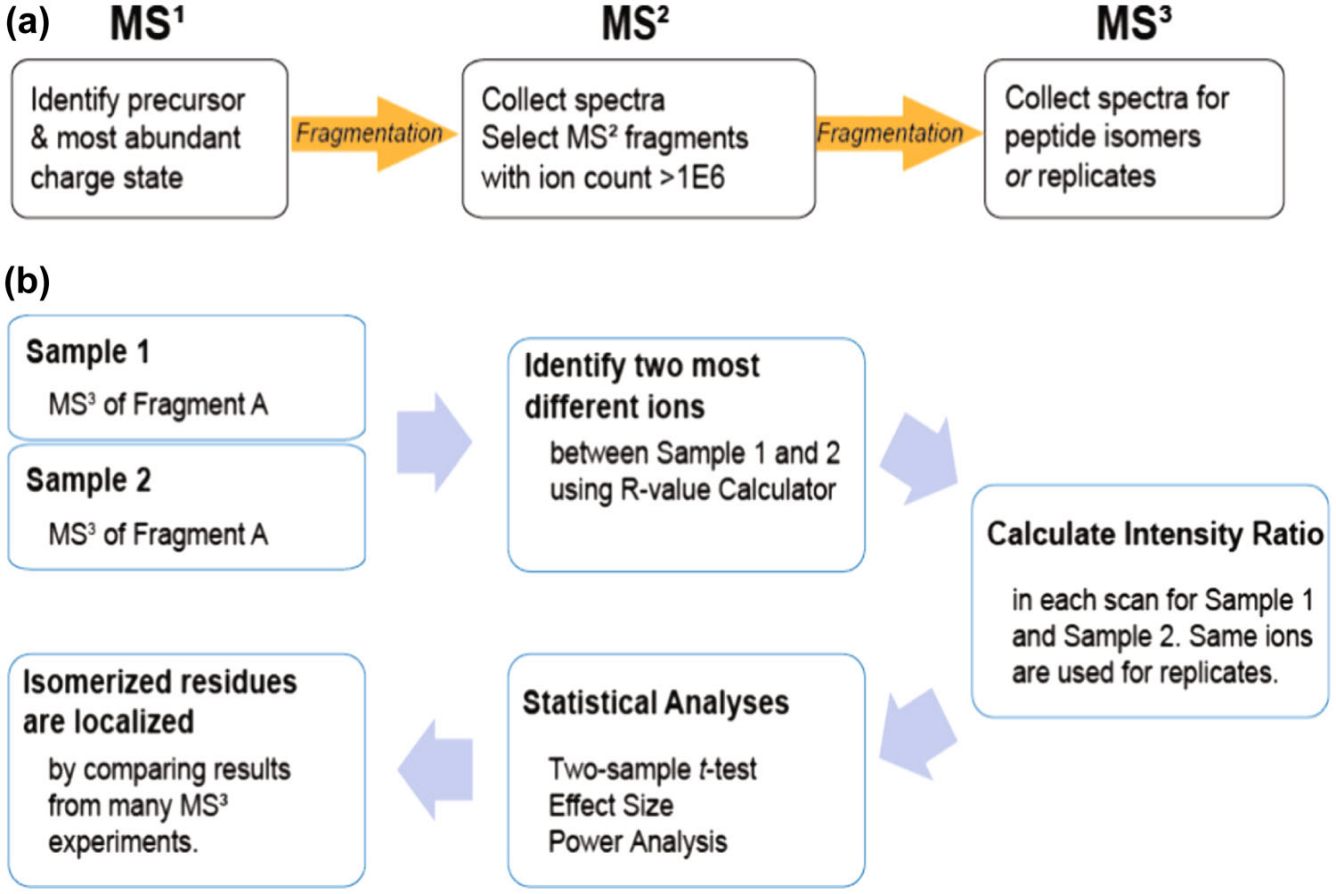
(a) Workflow for the site‐specific localization of isomerized residues using tandem MS. (b) Data Analysis process for site‐specific localization of isomerized residue using MS3 spectra. Figure adapted from H. T. Wu, Van Orman, and Julian (2024). [Color figure can be viewed at wileyonlinelibrary.com]

**Figure 4:**
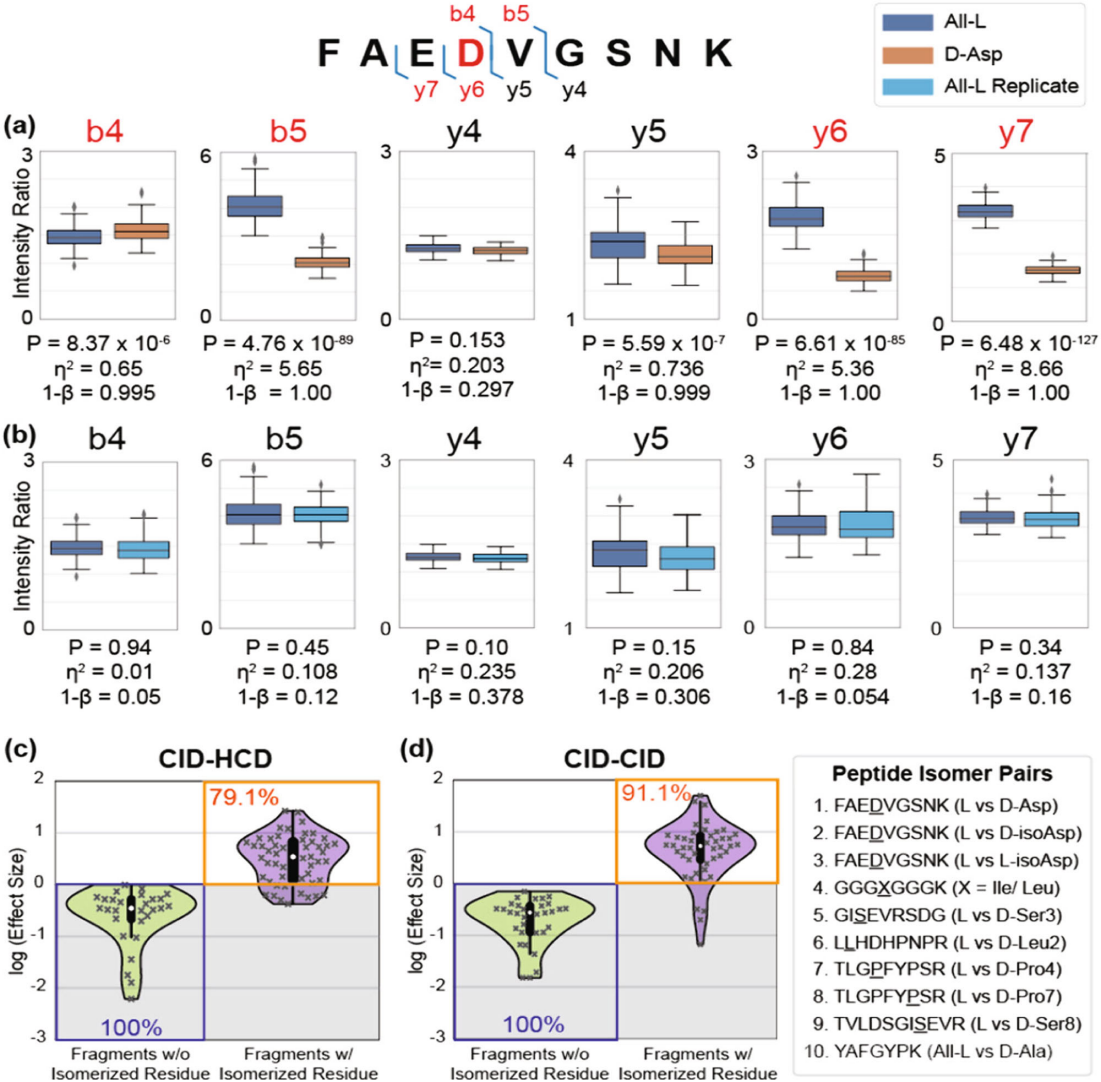
Localization of d‐Asp in [FAEDVGSNK + 2H]2 + . The fragment ions containing the isomerized residues are labeled in red. (a) The intensity ratios of the most different MS3 fragments between peptide isomers. All‐L and D‐Asp are plotted along with their corresponding statistical analyses for each fragment ion. (b) No statistically significant differences were observed in All‐L replicates. Collective results for 10 peptides using CID in MS2 followed by either (c) HCD or (d) CID in MS3. The log values (effect sizes) of the intensity ratios from two peptide isomers are categorized into fragments with or without the presence of isomerized residues (colored in green and purple). All fragment ions without the isomerized residue have a log10 effect size of <0, while most isomeric fragments have a log10 effect size of >0. Figure adapted from H. T. Wu et al. (2023). [Color figure can be viewed at wileyonlinelibrary.com]

While MS2 techniques are adept at distinguishing peptide isomers, they often do not locate the isomerization site. To address this limitation, the Julian group has developed an MS3 method specifically tailored for the site‐specific identification of isomerized residues (H. T. Wu, Van Orman, and Julian 2024). This method involves isolating fragment ions of peptide isomers using an ion trap, followed by fragmentation through collision‐induced dissociation (CID) and Higher‐energy collision dissociation (HCD) (Figure 3). They implemented an advanced statistical method to select pairs of MS3 fragment ratios that exhibit the most significant differences between isomers. By averaging multiple scans, this technique compensates for the variability in fragment ion intensity caused by the instrument, thereby enhancing the robustness of the approach. They confirmed significant differences in the MS3 fragment ion ratios of fragments containing the isomerized residues (Figure 4) through various statistical tests, including the two‐sample *t*‐test, effect size, and power analysis. The workflow for this MS3 strategy is detailed in Figure 3, offering a comprehensive guide for its application and further exploration in peptide isomerization studies.

### 2.3 MALDI‐MS Methods

MALDI MS is commonly used for rapid peptide identification and analysis of peptides in single cells and tissues including mapping the spatial distribution of the peptides in tissues by MALDI‐MS Imaging (Chan‐Andersen et al. 2022; Croslow, Trinklein, and Sweedler 2024; Rubakhin, Romanova, and Sweedler 2022; Xie et al. 2024) compared to ESI, the ability of MALDI to differentiate peptide isomers would improve analytical throughput and facilitate the mapping of the spatial distribution of peptide isomers in a tissue. Fragmentation techniques employed in MALDI‐MS such as post source decay (PSD), CID and PD can produce diagnostic peaks for isoAsp identification as well differential fragment ion abundance for discriminating and identifying other isomeric peptides (Hui et al. 2021; Koehbach et al. 2016; Pekov et al. 2019). In fact, CID and PSD promotes cleavages on the C‐terminal side of aspartic acid due to the formation of a seven membered hydrogen bond ring between the side of aspartic acid and the backbone C‐terminal amide oxygen, which is known as the D‐effect. Meanwhile the side chain of isoAsp cannot undergo such interaction hence not much fragmentation is observed for the isoAsp residues. Leveraging this effect various groups have quantified the levels of isoAsp using MALDI‐MS. Pekov and co‐workers used the fragment ion intensity ratios of b7 and b23 to quantify the amount isoAsp7 in Aβ‐amyloid (1‐41)(Pekov et al. 2019). Also, researchers from Amgen and the Loo group employed the D‐effect to quantify the amount of isoAsp in the complementary determining region of monoclonal antibodies (Hui et al. 2021). They noted the C‐trap dissociation or D‐effect is more profound in fragmentation of single charged species making the method more suitable for MALDI‐MS.

Our group also utilized MALDI‐TOF/TOF operating in LIFT mode with collisional activation by CID to quantify the amount of D‐tryptophan containing form of the cardioactive peptide NWF‐amide and other DAACPs in single neurons. The difference in stability of complexes formed between metal and peptide diastereomers can result in the generation of different fragmentation profiles that can be used to discriminate peptide epimers. Thus, metal addition can enhance peptide isomer discrimination. For example, the chiral recognition factor value of Cobalt adducts for FMRF‐amide peptide isomers increased by twofold compared to the protonated species (Bai, Romanova, and Sweedler 2011).

## 3 Ion Mobility Methods

Isomerized residues in peptides can significantly alter their three‐dimensional structures, consequently affecting their collisional cross‐section (CCS). Gas phase structural variation in isomers enables the separation of peptide isomers using ion mobility spectrometry (IMS) techniques, both linear and nonlinear (Harvey, MacPhee, and Barran 2011; Jeanne Dit Fouque et al. 2017; Shvartsburg et al. 2011, 2021; Q. Wu et al. 2020). Although Drift Tube IMS (DTIMS) has limited resolving power dependent on the tube length, it has been effectively used for differentiating peptide isomers (Harvey, MacPhee, and Barran 2011; Li, Delafield, and Li 2020; C. Wu et al. 2000). For instance, the Bowers research group achieved separation of Leu‐enkephalin (YAGFL and YdAGFL) peptide isomers using a 2‐meter long DTIMS instrument, which provided enhanced resolution not yet available in commercial DTIMS models. Additionally, the separation efficiency of DTIMS was improved by using different buffer gases, with helium proving to be the most effective (Kemper, Dupuis, and Bowers 2009).

Traveling‐Wave Ion Mobility Spectrometry (TWIMS), with a resolving power of approximately 50 on the CCS scale, has been employed to achieve partial resolution of peptide isomers. The Waters Synapt G‐2 instrument, which integrates TWIMS, features an MS/MS setup preceding IMS. This configuration was used by the Li group for the site‐specific identification of isomerized residues in peptides (Jia et al. 2014). They successfully localized D‐tryptophan, D‐alanine, and D‐phenylalanine residues within melanocyte‐stimulating hormone, deltorphin, and achatina peptides, respectively. Their approach involved chromatographically separating the isomers, subjecting them to CID, and then IMS analysis of the fragment ions. The precise locations of isomerized residues were determined by comparing the arrival time distribution of the fragment ions from each peptide epimer. However, due to the limited resolving power of the TWIMS, some isomeric fragments remained unresolved.

To address limitations in TWIMS resolution, Smith and colleagues introduced a new TWIMS‐based technique with an enhanced resolving power of approximately 350, known as the Structure for Lossless Manipulation (SLIM)(Deng et al. 2016). This advanced approach allowed for the complete resolution of all four amyloid Aβ‐42 peptide (H**D**SGYEVHHQK) isomers, including L‐isoAsp, D‐isoAsp, and D‐Asp. This innovation marks a significant advancement in the field of ion mobility spectrometry, offering greater precision in the analysis of peptide isomers.

**Figure 5:**
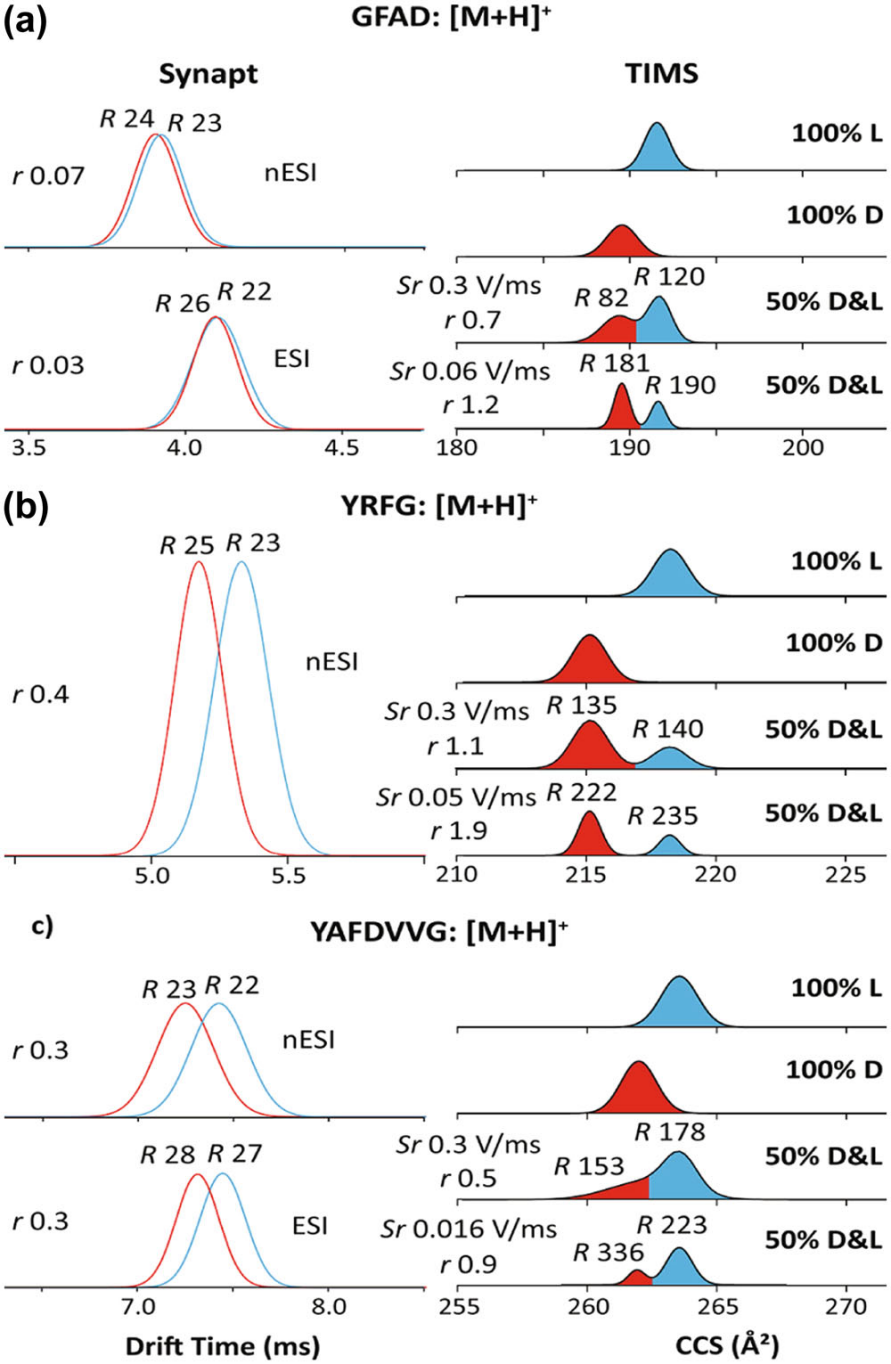
Analysis of peptide epimers by TWIMS and TIMS. IMS spectra by TWIMS (Synapt) (left) and TIMS (right) for small, protonated peptides (a) GFAD, (b) YRFG, and (c) YAFDVVG. The epimers are colored in blue (L) and red (D). The TIMS spectra for mixtures employed different scan rates Sr as marked. The resolving power (R = R = Ω/w) and resolution (r = 1.18*(Ω2 − Ω1)/(w1 + w2)) are indicated on the mobilograms. Figure was adapted from Jeanne Dit Fouque et al. (2017). [Color figure can be viewed at wileyonlinelibrary.com]

Trapped Ion Mobility Spectrometry (TIMS) is a linear IMS technique that utilizes an electric field gradient and a moving buffer gas to separate ions by size, mass, and charge. In TIMS, mobility separation is achieved by sequentially scanning the voltage to elute ions based on their size‐to‐mass ratio. The trajectory of ions can be adjusted in a TIMS device by varying the voltages, thereby creating different electric field gradients (Dodds and Baker 2019; Michelmann et al. 2015). This capability allows TIMS to achieve a resolution of approximately ~10–400 (K. J. Adams et al. 2016) positioning it as one of the high‐resolution IMS techniques. TIMS has been effectively used to differentiate several DAACPs from their all‐L isomers (Jeanne Dit Fouque et al. 2017; Mast, Checco, and Sweedler 2020). At a slow scan rate defined as the voltage range divided by the ramp time, baseline resolution was achieved for YdRFG/YRFG peptide isomers, with a CCS difference of 1.1%. In contrast, only partial resolution was observed for the GdFAD/GFAD peptide, which has a smaller CCS difference of 0.6% (Jeanne Dit Fouque et al. 2017) (Figure 5). Additionally, the incorporation of metal auxiliaries to form potassium adducts enhanced the resolution of isomers of the peptide “WKYMVM,” which could not be resolved in its protonated form even at very slow scan rates (Jeanne Dit Fouque et al. 2017). This adaptation demonstrates TIMS's flexibility and enhanced capability in resolving subtle structural variations among peptide isomers.

By integrating capillary electrophoresis with TIMS‐MS, our group successfully identified and differentiated isomers of the pleurin2‐peptide found in the cerebro‐pleural ganglion connectives of *Aplysia californica* (Mast et al. 2021). Additionally, we have recently developed a novel untargeted method that combines CID and TIMS for site‐specific localization of isomerized residues, including D‐amino acids and isoAsp, in several peptides (yet to be published).

**Figure 6:**
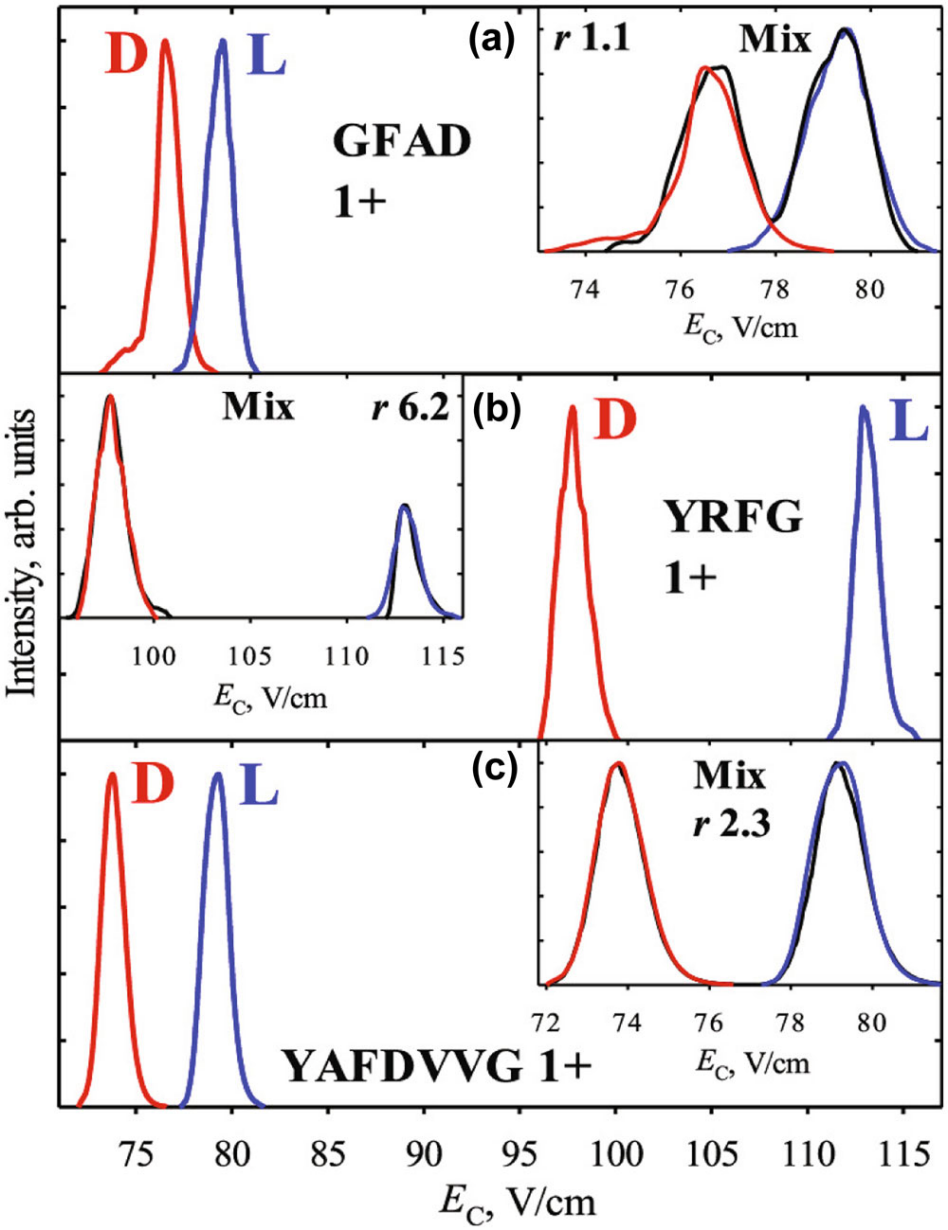
Normalized FAIMS spectra for the d‐ and l‐epimers of (a) GFAD (b) YRFG, and (c) YAFDVVG at z = 1. The data for isomeric mixtures (black trace) and scaled constituents (red and blue) are in the insets; the r values are marked. Figure is adapted from Berthias, Baird, and Shvartsburg (2021). [Color figure can be viewed at wileyonlinelibrary.com]

Field Asymmetric IMS (FAIMS) a nonlinear IMS technique, which exhibit high orthogonality to MS relative to linear IMS methods (Cooper 2016; Sweet et al. 2022), demonstrated enhanced capability in the differentiation of DAACPs and IACP from their diastereomers. Research conducted by the Shvartsburg group illustrated that FAIMS could achieve baseline resolution for peptides such as YRFG/YdRFG, YdAFDVVG/YAFDVVG, and GFAD/GdFAD isomeric pairs (Figure 6) (Berthias, Baird, and Shvartsburg 2021). In a comparative analysis, FAIMS successfully resolved all 20 peptide epimers tested, surpassing the performance of TIMS, which resolved only 17. The superior resolving capacity of FAIMS is attributed to its sixfold greater orthogonality to MS in comparison to TIM (Berthias, Baird, and Shvartsburg 2021).

Moreover, the identification of isoAsp residues in therapeutic peptides was successfully carried out using cyclic IMS (cycIMS) by Gibson K. and collaborators. This team identified the sites of isoAsp residues by analyzing cycIMS data of the fragment ions from each isomeric pair. By comparing the arrival time distributions of fragment ions and monitoring shifts in drift times for those containing the isomers, they effectively confirmed the sites of isomerization (Gibson et al. 2022).

Shvartsburg and colleagues utilized a combination of advanced IMS techniques, including cycIMS, TIMS, and FAIMS, to successfully resolve the largest documented DAACP. This peptide, consisting of 72 amino acid residues, is a member of the crustacean hyperglycaemic hormone (CHH) family. Their work highlights the potential of collision‐induced unfolding (CIU) as a tool to distinguish between DAACPs and their epimeric counterparts. The ion mobility methods employed in this study demonstrate promising capabilities in resolving complex peptide epimers, which are otherwise challenging to distinguish using traditional mass spectrometry alone. These techniques enable higher resolution and structural insight into peptide epimers, offering a significant advancement in the field of peptide analysis (Thurman et al. 2024).

## 4 Chromatographic Methods

Due to the variations in their three‐dimensional structures, peptide diastereomers exhibit different interactions with stationary phases, such as C‐18 and chiral columns. These differences in interaction are important because they lead to the diastereomers eluting at distinct retention times during chromatography (Maeda et al. 2015; Winter, Pipkorn, and Lehmann 2009). Split eluting peptide peaks in chromatography have been instrumental as a marker for identifying isomerized residues within peptides (Bai, Sheeley, and Sweedler 2009; Livnat et al. 2016). Among various techniques, reverse‐phase (RP) chromatography stands out as one of the most effective methods for the separation of peptide isomers (Jansson 2018). Coupling RP‐chromatography with enzymatic screening processes has facilitated the identification and study of specific DAACPs and IACPs (Livnat et al. 2016; Mast, Checco, and Sweedler 2020; Tao and Julian 2014).

Isomerization in peptides can also influence the pKa values of ionizable groups, which in turn affects the isoelectric point of the peptide. This variation in the isoelectric point is significant because it can change how peptides behave under different pH conditions. Consequently, capillary electrophoresis, which is adept at separating molecules based on their charge‐to‐size ratio, has been employed effectively for the separation of peptide epimers, particularly in analyses involving small volumes (Mast et al. 2021).

## 5 Enzymatic Enrichment and Labeling of Zero‐Dalton PTMs

The enzymatic activities of proteolytic enzymes and peptidases on peptides are significantly influenced by the presence of isomerized residues within the peptides (Livnat et al. 2016; Silzel et al. 2022). These modifications can either slow down or accelerate the degradation rate of peptides containing DAACP and IACP. Capitalizing on the increased resistance of peptides with isomerized residues to enzymatic degradation, various research groups have developed enrichment strategies to facilitate the identification of isomeric peptides (Livnat et al. 2016; Mast, Checco, and Sweedler 2020, 2021; Ni et al. 2010; Silzel et al. 2022). The enzyme endoproteinase AspN, which typically cleaves at the N‐terminal side of aspartic acid residues, exhibits a reduced cleavage rate at isoAsp residues. A method involving the treatment of a peptide mixture containing both isoAsp and L‐Asp isomers with AspN followed by Electron Transfer Dissociation (ETD) analysis to detect C* + 57 and Z‐57 diagnostic peaks has been reported (Ni et al. 2010). This analysis revealed complete digestion of the Asp‐containing peptide, whereas the isoAsp‐containing peptide remained undigested. The technique effectively confirmed the presence of isoAsp residues in amyloid‐beta (Aβ‐42) and cytochrome‐C peptides.

Protein isoaspartyl carboxyl‐methyltransferase (PIMT) catalyzes the conversion of isoAsp to aspartate by selectively methylating the isoAsp residue using S‐Adenosyl Methionine as the methyl donor (R. Desrosiers & Fanelus n.d; Zhu et al. 2006). This enzymatic activity has been utilized to analyze isoAsp levels in biological samples (Alfaro et al. 2008; Liu et al. 2012; Silzel, Lambeth, and Julian 2022). The methylation induces a mass shift of +14 Da, which should theoretically aid in the site‐specific localization of isoAsp by tandem MS. However, achieving this is challenging due to the low stability of the methylated isoAsp (Alfaro et al. 2008), which tends to cyclize into a succinimide intermediate. Despite these difficulties, we have developed an approach that can capture and detect methylated forms of isoAsp‐containing peptides by carefully controlling the enzyme kinetics. Using our refined PIMT assay and CID, we successfully pinpointed isoAsp residues in rats’ neuropeptides extracted from the hypothalamus and spinal cord (manuscript in preparation).

Also, the localization of isoAsp residues was achieved by employing an excess of hydrazine to react with the succinimide intermediate produced during the PIMT reaction. This reaction results in a mass shift of +14 Da. Tris‐base was used as a nucleophile for the precise localization of isoAsp residues. The peptide succinimide intermediate reacts with Tri's base, forming a conjugate that enhances chromatographic separation and introduces a significant mass shift of +103 Da. The technique was successfully applied to identify isoAsp residues in a tryptic digest of crystallin from eye lenses (Silzel, Lambeth, and Julian 2022).

Aminopeptidase M (APM) is an exopeptidase that catalyzes the removal of amino acids from the N‐termini of peptides. The isomerization of amino acid residues near the N‐termini enhances peptide resistance to APM degradation, making APM a useful tool for the enrichment of peptides containing D‐amino acids. Once enriched, these peptides are typically isolated and analyzed for the presence of D‐amino acids by acid hydrolysis and amino acid analysis (Livnat et al. 2016; Mast, Checco, and Sweedler 2020). This approach has been effectively employed by our group for the identification of several DAACPs in metazoans including the sea slug *A. californica*. Here LC‐MS analysis of the APM‐treated extract revealed the degradation of all ‐L peaks previously separated by LC, whereas the DAACPs form was still present. Synthetic standards were used to further confirm the site of isomerization in Pleurin‐1‐3, Ip‐1‐2, and FMRFGF‐amide peptides (Mast, Checco, and Sweedler 2020).

## 6 Conclusion and Future Perspective

Recent advancements in high‐throughput mass spectrometry and bioinformatic technologies have significantly enhanced peptidomics analysis capabilities (Hellinger et al. 2023). Contemporary peptide libraries now often integrate machine learning algorithms to predict various characteristics of peptides, such as fragment ion abundance distribution, ion mobility, and the CCS (C. Adams et al. 2024; Meier et al. 2021). Despite these technological advances, the incorporation of peptide diastereomer identification into routine peptidomics or proteomic workflows remains limited. Currently, the methods employed for identifying peptide isomers are predominantly targeted and can be labor‐intensive, posing substantial challenges in the detection of peptide diastereomers.

While enzyme activities such as L/D isomerase have been detected in various tissues such as mouse hearts, rat lungs, brains, spinal cords, and bovine lungs, the identification of DAACPs in these species is still forthcoming (Andersen et al. 2024). IsoAsp formation has primarily been associated with aging due to its prevalence in age‐related diseases like Alzheimer's and cataracts (Hooi and Truscott 2011; J. Wang et al. 2023). However, new roles of IsoAsp in biological processes, including activating extracellular matrix proteins, initiating integrin binding, and enhancing cellular adhesion to biomolecules, have been reported (Akbey and Andreasen 2022; Jacob et al. 2016). Observations of high IsoAsp levels in young PIMT‐deficient mice also suggest that the formation of IsoAsp extends beyond merely age‐related phenomena (Yamamoto et al. 1998). For these reasons, it is essential to develop new tools and methods to probe these modifications. We expect that as these tools become used, we will see a large increase in the reported prevalence of these modifications.

A significant challenge in advancing our understanding of these zero‐dalton PTMs in endogenous peptides and proteins is the lack of untargeted analytical and computational techniques capable of identifying such modifications. Several ion mobility spectrometry (IMS) and tandem mass spectrometry, along with chromatographic methods, have been demonstrated to differentiate peptide diastereomers effectively (Jia et al. 2014; Mast, Checco, and Sweedler 2020; Shvartsburg et al. 2011; H. T. Wu, Van Orman, and Julian 2023, 2024), it is evident that existing proteomic and peptidomic datasets contains overlooked information relevant to DAACPs and IACPs. To unlock this potential, there is a pressing need for the development of analytical tools that specifically target fingerprints of isomerized residues. Such tools would look for indicators such as peptides with split eluting chromatographic peaks, mobility differences between such peaks, and chiral recognition factors for potential isomeric peaks.

Moreover, the design of advanced computational and machine learning strategies that include peptide epimers in‐silico sequencing methods could significantly improve the detection and identification of zero‐dalton PTMs. The creation of a comprehensive predictive CCS library that accounts for isomerized residues would further enhance the identification capabilities of DAACPs and IACPs if seamlessly integrated into bioinformatics platforms for peptide identification. These new analytical capabilities would provide novel insights into the complex dynamics of protein and peptide modifications and interactions in biological systems.

## Author Contributions

**Samuel Okyem:** conceptualization, writing–original draft, writing–review and editing. **Jonathan V. Sweedler:** conceptualization, Resources, writing–review and editing. The authors contributed equally to the writing of this review article.

## Conflicts of Interest

The authors declare no conflicts of interest.
